# Exploring communication and implementation challenges of the HIV/AIDS policy change to test-and-treat-all in selected public health facilities in Lusaka District, Zambia

**DOI:** 10.1186/s43058-023-00430-6

**Published:** 2023-05-12

**Authors:** Constance Simooya, Adam Silumbwe, Hikabasa Halwindi, Joseph Mumba Zulu, Selestine Nzala

**Affiliations:** 1 Department of Health Policy and Management, School of Public Health, University of Zambia, Lusaka, Zambia; 2 Department of Epidemiology and Global Health, Umeå University, 901 87 Umeå, Sweden; 3 Department of Community and Family Medicine, School of Public Health, University of Zambia, Lusaka, Zambia; 4 Department of Medical Education, School of Medicine, University of Zambia, Lusaka, Zambia

**Keywords:** Communication, HIV/AIDS, Implementation, Policy change, Test-and-treat-all, Zambia

## Abstract

**Background:**

The World Health Organization has promoted a shift towards the test-and-treat-all strategy to accelerate the elimination of HIV/AIDS. Zambia was one of the early African countries to adopt this strategy as the policy change was officially announced on national television by the republican president on 15th August 2017. This study explored the communication and implementation challenges of the HIV/AIDS policy change to test-and-treat-all in selected public health facilities in Lusaka District, Zambia.

**Methods:**

A qualitative case study design was employed with a purposeful sample of policy makers, international partners, National AIDS Council representatives, health facility managers, and frontline health providers in selected tertiary, secondary and primary health facilities in the Lusaka District, Zambia. Thematic data analysis was performed using NVivo 12 Pro software.

**Results:**

In total, 22 key informant interviews and 3 focus group discussions were conducted. The government relied on formal and informal channels to communicate the test-and-treat-all policy change to health providers. Whilst HIV policy changes were reflected in the National HIV/AIDS Strategic Framework, there was little awareness of this policy by the frontline providers. The use of informal communication channels such as verbal and text instructions affected health providers’ implementation of the test-and-treat-all. Electronic and print media were ineffective in communicating the test-and-treat-all policy change to some sections of the public. Top-down stakeholder engagement, limited health worker training, and poor financing negatively affected the implementation of the test-and-treat-all policy change. Acceptability of the test-and-treat-all policy change was shaped by positive provider perceptions of its benefits, limited sense of policy ownership, and resistance by the non-treatment-ready patients. Furthermore, unintended consequences of the test-and-treat-all policy change on human resources for health and facility infrastructure were reported.

**Conclusion:**

Effective test-and-treat-all policy change communication is vital for successful policy implementation as it enhances interpretation and adoption among health providers and patients. There is a need to enhance collaboration among policy makers, implementers and the public to develop and apply communication strategies that facilitate the adoption of the test-and-treat-all policy changes to sustain gains in the fight against HIV/AIDS.

Contributions to the literature

- This study contributes to the limited literature on the communication of policy change and policy implementation. Communication of policy change is critical in ensuring that policy intentions find the correct expressions in the health systems.
- Effective health policy change communication is vital for successful policy change implementation as it enhances understanding and subsequent adoption of the policy.
- There is a need to enhance collaboration among policy makers, partners, implementers and the public in developing and applying communication strategies that facilitate the quick adoption of health policy changes.

## Background

Globally, approximately 36.9 million people are living with HIV/AIDS [1]. Sub-Saharan Africa carries a disproportionate burden of HIV/AIDS, accounting for more than 70% of the global burden [2]. In Zambia, HIV prevalence is estimated to be 11% among adults and 15% among children, and is higher among urban compared to rural residents (9.8% vs 5.0%) [3]. Furthermore, there has been an increase in HIV prevalence among urban young men from 3.7% in 2001–2002 to 7.3% in 2013–2014 [4], and 9.4 million Zambians do not know their HIV status [5].

Over the years, Zambia has adopted various policies to address HIV/AIDS. These include the HIV/AIDS voluntary counselling and testing strategy which was expanded to include the prevention of mother-to-child transmission (PMTCT) in 1999. In 2002, the government created the National HIV/AIDS/ STI/TB Council (NAC) whose mandate was to oversee HIV, sexually transmitted infections, and tuberculosis policies and programmes. The creation of NAC led to the introduction of many HIV policies such as the HIV work policy, counselling and testing (C&T) and HIV post-exposure prophylaxis for exposed health providers and clients [6]. In 2013, diagnosis, counselling and testing (DCT) was introduced where all admitted patients were mandated to undergo HIV testing and counselling [7]. Further changes to the HIV policy saw the mother to child transmission evolve from voluntary PMTCT to option A, B, compulsory PMTCT option B + (drug treatment regimens for HIV-positive pregnant women and their infants) to now the elimination of mother-to-child transmission (EMTCT) [8].

In line with the World Health Organization’s call to zero-rate new HIV infections and mortality, Zambia adopted the test-and-treat-all policy or the “90–90–90 strategy” in 2017, a shift from voluntary counselling and testing. This policy entails that 90% of people living with HIV know their status, 90% of people living with HIV who know their status access treatment and 90% of those living with HIV who are receiving treatment have suppressed viral load [9]. The targets 90/90/90 aim to control and eliminate HIV infection by 2030. Through the test-and-treat-all policy, a person who tests HIV-positive is immediately started on treatment. Before this policy, people living with HIV had to wait for their CD4 count to drop to a particular threshold before starting treatment. According to the Joint United Nations Program on HIV/AIDS, progress has been made since the adoption of the test-and-treat-all policy in Zambia [10].

Despite the progress in HIV/AIDS policy in Zambia, HIV/AIDS still remains one of the greatest threats to population health [3]. The changes in HIV/AIDS policy have come with some implementation successes and challenges. Contextual factors such as stigma and discrimination, negative traditional and religious beliefs, disease epidemiology, weak leadership, inadequate  human resources, poor funding, political and economic challenges have affected the success of HIV/AIDS policy changes [11]. Other implementation gaps to HIV/AIDs policies relate to poor retention on treatment, in availability of nutritional supplements, and low uptake of isoniazid preventive therapy [12, 13].

Whilst the above implementation gaps in HIV/AIDS policy changes are widely documented, how these policy changes are communicated among policy makers, health providers and patients is understudied. Studies show that the manner in which health policy changes are introduced and communicated in a health system can impact implementation success [14]. Communication of policy changes is critical to ensure policy intentions find the correct expression in local health systems [15, 16]. Therefore, understanding how the HIV/AIDS policy change was communicated contributes to enhance its implementation.

In Zambia, HIV/AIDS policy is governed and communicated through the National HIV/AIDS Strategic Framework developed by the NAC which is updated every 4 years [17]. However, the change to the HIV test-and-treat-all policy was first announced on national television by the Zambian president who disclosed that this was official government policy [18]. Though there was strong opposition to mandatory HIV testing among stakeholders, the policy was subsequently adopted across the health system.

This study explored the communication and implementation challenges of the HIV/AIDS policy change to test-and-treat-all in selected public health facilities in Lusaka District, Zambia.

## Methods

### Study design

A qualitative case study was undertaken. The case study was appropriate because it allowed the researcher to conduct an in-depth exploration of the health policy change communication and implementation process [19, 20]. The case study comprised the test-and-treat-all policy and the stakeholders who were involved in its communication and implementation in Lusaka District. The study uses the consolidated criteria for reporting qualitative research (COREQ) guidelines to describe key aspects of the research team, study methods, study setting, findings, analysis and interpretation (additional file).

### Study site

The study was conducted in the Lusaka District, the capital city of Zambia. It was purposefully selected due to the role it plays in policy leadership and oversight. Most high-level HIV policy institutions including the Ministry of Health (MoH), National AIDS Council and other international partner institutions are found in the capital. Policy changes are mostly decided in Lusaka and then communicated to all other provinces. Three health facilities at primary, district and tertiary level were selected as study sites based on the recommendation of the MoH policy makers and district health office leaders. Further, these facilities were reported to have a high influx of HIV/AIDS patients on a daily basis [21].

### Sampling criteria and participant recruitment

Purposive sampling was used to select participants relevant to the study at different levels of health care system—including primary, district, tertiary and national. Snowballing was used to recruit policy makers and identify other key players in the test-and-treat-all policy process. We were guided by the HIV/AIDS task force lead at the national level, to identify international partners and health facilities that were engaged in the communication and implementation of the test-and-treat-all policy. The study participants were categorized as frontline health providers, health facility managers, international partners and policy makers. The principle of theoretical saturation which is the point at which no new data nor insights emerge from additional interviews guided the number of participants we interviewed for each category [22].

### Data collection

The qualitative data were collected using key informant interviews and focus group discussions over a period of 5 months from November 2019 to March 2020. Phone calls were made to potential participants to schedule face-to-face interviews. Where the participant was unable, we conducted the interviews using Zoom. Four interviews were done via Zoom. Both the key informant interviews and focus group discussions were conducted away from the participant’s workplace to accord them the freedom to express themselves. Data were collected using audio recorders and stored on a password-secured laptop computer. Notes were also taken during the data collection. Interview and focus group discussion guides were used to stir the discussions. The questions in both guides were phrased according to the category of participants to elicit context-specific information regarding HIV policy change, communication, support and implementation. All the interviews were conducted in English by the first author.

### Data analysis

Thematic analysis approach was used in this study [23]. The audio-recorded interviews were transcribed verbatim using the Express Scribe v8.26 software. Firstly, data were read to develop codes that were identified from participant expressions, phrases and topics during the interviews and focus group discussions. The second phase comprised the identification of patterns, similarities and variations in the codes to develop the main and sub-themes. Inductive coding was used for this study [24]. A master codebook with thematic definitions agreed upon by the research team was developed (Table 3). Two researchers CS and AS double coded the all transcripts using NVivo 12 Pro software. The final coding reports were then shared with the research team for further analysis and reporting.

### Ethical considerations

Approvals to conduct the study were sought from the University of Zambia Biomedical Research and Ethics Committee [reference number 277–2019] and National Health Research Authority. Permission to interview the study participants was provided by the provincial and district health office,  and health facilities' managers. This study had minimal to no risks. Voluntary written informed consent from individual participants was obtained. To ensure confidentiality, the participant names were anonymized during the interviews/discussions and data analysis by using participant identification codes as opposed to their actual names.

## Results

### Key informant interviews

A total of 22 key informant interviews were conducted across Lusaka, with key state and non-state actors in HIV/AIDS policy change (Table 1). The key informants comprised policy makers from MoH, NAC managers, Lusaka District Health Office (LDHO), HIV/AIDS programme managers, international partners and health facility managers from the primary, secondary and tertiary health care facilities involved in HIV/AIDS management and policy communication.

**Table 1:** Key informant interview participants

Participants	Number	Institution
Policy-makers—health policy department	4	MoH
Policy-makers—public health clinical	3	MoH
Programme manager HIV partners	1	PEPFAR
National HIV/AIDS.STI/TB council managers	3	NAC
Health facility managers	2	Tertiary health facility
District health office facility manager	1	LDHO
District health office HIV/AIDS programme managers	2	LDHO
Health facility managers—clinical	3	Secondary health facility
Health facility managers	3	Primary health care
**Total**	**22**	

### Focus group discussions

Three focus group discussions were conducted with frontline healthcare providers involved in the implementation of the test-and-treat-all policy change at primary, secondary and tertiary healthcare facilities. This was done to capture rich and diverse perspectives regarding the HIV test-and-treat policy change among the frontline providers. Each focus group discussion had 7–11 participants per session, lasted between 45 minutes and 1:30 hours, and the participants comprised HIV/AIDS clinicians and psychosocial counsellors. A cumulative number of 26 frontline providers participated in the focus group discussions (Table 2).

**Table 2:** FGD participant categories

Level of healthcare facility		Participant category		Total
Tertiary	1	Doctors	1	8
		Nurses	5	
		Psychosocial counsellors	2	
Secondary	1	Doctors	1	11
		Nurses	7	
		Psychosocial counsellors	3	
Primary	1	Nurses	5	7
		Psychosocial counsellors	2	
Total	**3**			**26**

Our findings on the communication and implementation challenges of the HIV/AIDS policy change to test-and-treat-all in Zambia are presented based on the policy makers and health provider perspectives. Emerging themes from the data are organized according to the two main thematic areas of communication strategy and implementation challenges of the HIV/AIDS test-and-treat-all policy change (Table 3). Although data were collected from various participants, no major differences in the perspectives were noted. Any views specific to a particular participant category are reported as such within the manuscript.

**Table 3:** Main themes and sub-themes

Main theme	Sub-theme	Sub-theme
1.Communication strategy of the HIV/AIDS policy change to test-and-treat-all	•National HIV/AIDS Strategic Framework and its role in policy change communication	•Providers lack awareness of the framework
	•Communication channels of HIV policy change to test-and-treat-all	•Formal and informal notification of health providers regarding HIV policy change to test-and-treat-all •The role of the media (digital and print) in communicating the HIV policy change to test-and-treat-all to the public and health providers
2.Implementation challenges of the HIV/AIDS policy change to test-and-treat-all	•Top-down stakeholder engagement in test-and-treat-all policy change •Limited healthcare worker training for implementation of test-and-treat all policy change •Inadequate materials and resources to support test-and-treat-all policy change •Limited financing of test-and-treat-all policy change	
	•Acceptability of the test-and-treat-all policy change	•Positive perception of the potential benefits of HIV policy change to test-and-treat-all among providers •Limited sense of ownership of HIV/AIDS policy change to-test and-treat all among providers •HIV patient’s resistance of policy change to test-and-treat-all
	•Unintended consequences of the HIV/AIDs policy change to test-and-treat-all	•Increased demand for human resources in health facilities •Changes in work schedules •Increased working hours •Infrastructure adjustment to accommodate expanded HIV services

#### Communication strategy of the HIV/AIDS policy change to test-and-treat-all

The participants had various perspectives regarding the communication strategy of the policy change to test-and-treat-all. Two themes emerged from the data. Firstly, the national HIV/AIDS policy governance strategic framework and its role in policy change communication. Secondly, the communication channels/mechanisms used to create awareness of the test-and-treat-all policy change to the health providers providers and the public.

##### National HIV/AIDS Strategic Framework and its role in policy change communication

The policy makers disclosed that government has an outlined policy communication strategy that is operationalized through the HIV/AIDS strategic framework for 2017–2021, which provides for coordination and management of the HIV/AIDS policy response in Zambia. The coordination of the HIV/AIDS policy takes place at various levels of the health system and seeks to align national priorities with stakeholder efforts.

There is a communication strategy guided by the HIV/AIDS strategic framework on how each area of the health system is informed about HIV/AIDS policy changes from the facilities to community leaders all the way to the people living with HIV (KII 02, Policy maker, MoH).

However, the frontline health providers revealed that the communication strategy for HIV/AIDS policy change to test-and-treat-all adopted by the MoH was inadequate. They narrated that the change to the test-and-treat-all policy took them by surprise, as they had limited prior information. They felt that the policy change was hastily done without prior orientation and planning.

Even us, health workers, we were not ready for it [policy change]. They [Ministry of Health] said, just go test-and-treat-all. We felt sorry for the clients, but we had to explain that it was a government policy. So, we just had to do it (FGD 03, frontline health provider).

##### Communication channels of HIV/AIDS policy change to test-and-treat-all

**Formal and informal notification of health providers regarding HIV/AIDS policy change to test-and-treat all**

The policy makers stated that to communicate health policy, the MoH generates a detailed circular that is transmitted to all levels of the health system including primary care facilities. However, the frontline health providers indicated that this process seemed not to have been fully utilized when introducing the test-and-treat-all policy. Even when the above process was followed, the communication of the test-and-treat-all policy change delayed reaching the implementers and communities. Furthermore, some frontline providers stated that they were given verbal instructions via phone calls and informal messages about the change to test-and-treat-all before formal communication to the facilities.

So, policy ideally is communicated from the district, the district gets it from the province, province from the ministry through a circular. But with new technology, many are the times where you just see a WhatsApp message, oh! This is from the ministry. But we always wait for official communication from the district before we act on anything (KII 07, Health facility manager).

**The role of the media (digital and print) in communicating the HIV policy change to test-and-treat-all to the public and health providers**

The policy change was first communicated on national television by the republican president who announced that HIV test-and-treat-all would be mandatory in government facilities. The presidential announcement signified the importance government attached to the policy change.

It was the first time that the president has had to announce HIV policy change on national television. This was usually done by the Minister of Health (KII 14, Health manager).

Some frontline providers admitted that they first got to learn about the policy change during the presidential pronouncement as one of them expressed.

I personally got it from the TV that there is test-and-treat-all. There was no communication from management, am sure a good number of us got it from TV (FGD 01, frontline provider).

Following the presidential pronouncement, many media houses such as print, and radio intensified the dissemination of the test-and-treat-all policy change.

Dissemination was done using TV and radios, but it’s like many people did not understand what it [test-and-treat-all policy] meant. A lot of people were still in denial despite being told about that test-and-treat-all policy change. When it started it wasn’t easy (KII 08, District health manager).

The policy makers mentioned that newspapers, brochures and posters were used to disseminate the test-and-treat-all policy change to the public. However, the frontline health providers felt that the distribution of English print media did not reach population segments that were unable to read. They also thought using print media was ineffective for certain sections of society with special demographic features. For example, few people and mostly senior citizens buy newspapers. Furthermore, the health providers mentioned that late delivery of print media to both the health facilities and communities affected real-time communication of the HIV policy change to test-and-treat-all.

Again, what comes first is English posters, these our people cannot read. Even local language sometimes it becomes a political issue which language to use. The message on pictures sometimes needs to be explained. (FGD 09, frontline provider)

#### Implementation challenges of the HIV/AIDS policy change to test-and-treat-all

The participants highlighted several issues affecting the implementation of the policy change to test-and-treat-all after the presidential announcement and official adoption by the Ministry of Health. We describe participants’ views regarding stakeholder engagement, health provider training, commodities and supplies, financing, acceptability and the unintended consequences of the HIV/AIDS policy change to test-and-treat-all.

##### Top-down stakeholder engagement in the test-and-treat-all policy change implementation

The facility managers indicated that international partners in HIV/AIDS programming were engaged to support the test-and-treat-all policy change. For example, the United States President’s Emergency Plan for AIDS Relief (PEPFAR) and Centres for Disease Control and Prevention (CDC) played a critical role in providing resources to advertise and explain the HIV policy change to test-and-treat-all. Similarly, community-based organizations were also engaged to support the test-and-treat-all policy change.

The International partners including PEPFAR have been helpful in creating awareness regarding the test-and-treat-all. They have really come through to support where government has been unable by providing resources (K1106, facility manager).

However, the frontline providers felt that the engagement was top-down, and not much attention was paid to the communities given the sudden change in HIV/AIDS policy. They indicated that some patients who were unaware of the policy change were surprised at being put on treatment immediately after testing positive.

Look, whilst there have been efforts to engage various stakeholders. I think not much was being done to engage the patients themselves at the time of the policy change. It was difficult to convince patients to start treatment (FGD03, frontline providers).

##### Limited healthcare provider training for the implementation of test-and-treat-all policy change

The policy makers indicated that some facilities were selected to pilot the test-and-treat-all policy before national wide scale-up. However, some frontline providers stated that not much was known about this pilot, as it only covered a few selected staff and health facilities. Furthermore, the lessons from the pilot seemed not to have been widely disseminated to the other facilities. In addition, the frontline providers mentioned that they were varying levels of implementation of the test-and-treat-all in the facilities due to limited training in some instances.

Throughout the country the test-and-treat-all policy is being practiced. The variance in implementation may be there. For example, we have guidelines on how to test-and-treat but, in some facilities, it’s a bit difficult to implement because they may not have the training (KII 02 Policy maker).

In terms of training, before the roll out of the test-and-treat-all policy, I think not much happened. But I have attended training such as TB management and other programs. What I can say is that there was no specific training for the-test-and-treat-all policy(FGD, 5, frontline provider).

##### Inadequate materials and resources to support the test-and-treat-all policy change

The facility managers and providers explained that there was a varying supply of resources depending on the level of the health facility and the volume of patients. The frontline providers felt that majority of the health facilities did not receive adequate supplies and commodities to support the scale-up of the test-and-treat-all policy leading to frequent stock-outs of key supplies such as testing and urine kits. They indicated that most health facilities had limited resources to for initiating key baseline HIV investigations as one primary health facility manager narrated.

We were not supported…no, we were not supported. I can say for test-and-treat-all, the key things we were struggling with, and even now, are basics such as a urine stick. You want to have at least that dip stick as you are initiating the client (KII 07, Health facility manager).

However, through international partners’ support, key supplies and human resources were provided. One provider mentioned how international partners facilitated the acquisition of testing kits and recruitment of treatment counsellors to support the test-and-treat all policy change.

Through our partners like CDC and CIRDZ, they employed more peers as treatment supporters and counsellors. So, we received more counsellors and testing kits. So, we were able to do most of the activities (KII 11, frontline provider).

##### Limited financing of the test-and-treat-all policy change

The policy makers narrated that the funds committed to the scale-up of the test-and-treat-all policy change by the government were inadequate. They indicated that funding from international partners was critical to sustain the HIV policy change to test-and-treat-all. Some partners increased their funding commitments to the HIV response after the adoption of the test-and-treat-all policy. Further, partner financing support included remuneration of staff at the health facilities and availing resources for policy change monitoring and evaluation.

Zambia is divided into two parts. The northern region has six provinces which are supported by USAID. Then the others Lusaka, Eastern, Western, and Southern are supported by CDC. In each of these, they give funds to different organizations to support the HIV/AIDS test-and-treat-all in the facilities (KII 03 HIV International partner - manager).

However, both the policy makers and health providers agreed that it was unsustainable to have the majority of HIV services funded by international partners.

The partners are important to the HIV fight due to their huge financial commitments. For example, PEPFAR is literally funding our entire HIV program. While this may show that we have reliable partners, it’s also not sustainable for an entire country (KII 04, Health facility manager).

##### Acceptability of the test-and-treat-all policy change among providers and patients

They were both positive and negative sentiments towards the HIV/AIDS policy change to test-and-treat-all. Some emergent themes shaping the acceptability of the test-and-treat-all included the benefits of policy change, lack of sense of policy ownership by frontline providers and policy change resistance among HIV patients.

**Positive perception of the potential benefits of HIV/AIDS policy change to test-and-treat-all among health providers**

Both the facility managers and frontline providers explained that the test-and-treat-all policy was widely accepted among healthcare workers because it made HIV service provision easier. They thought early testing-and-treatment entailed putting the patients in a better position to avoid opportunistic infections and mortality. Being able to manage HIV in its early stages meant that future patient visitations to the facilities would be reduced. Furthermore, screening and checking procedures were simplified in the test-and-treat-all policy. A frontline health provider had this to say.

We have benefited because the test-and-treat-all has helped us to identify what might be disturbing a particular HIV patient and put them on the correct drugs. Two weeks later the patient recovers. These are patients who could have been in and out of the ward for a long time. But once you put someone on correct medicines, two weeks later, they are fit… (KII 08 DHO HIV program manager 02).

**Limited sense of ownership of HIV/AIDS policy change to test-and-treat all among health providers**

Some health facility managers and frontline health providers mentioned that they had a limited sense of ownership of the HIV/AIDS policy change to test-and-treat-all.

They perceived this policy change as an agenda by the Ministry of Health to impress international partners to attract more funding. In particular, the large number of partner organizations supporting the HIV policy change agenda made the health providers to question its ownership and intentions. One manager had this to say:

I had doubted the intentions of the test-and-treat-all policy change. Initially, it looked as though it was one of those policies we embraced as a country to attract funding for HIV programmes from the international community (KII 14 Health facility manager 01).

**Patient resistance of HIV/AIDS policy change to test-and-treat-all**

Both the facility managers and frontline health providers explained that when the test-and-treat-all policy was first rolled out to the public many community members resisted because they felt that it was imposed on them. They narrated that they had to convince the clients seeking HIV services to accept being tested and treated. Furthermore, the health providers revealed that some of the clients initiated on treatment were defaulting because they were not ready. One health provider manager stated.

It was difficult, and it is still difficult. We are struggling with the issue of retention into care, but are fighting hard by continuing with sensitization. Community peers follow some clients to just explain to them on the benefits of being on medication. But I cannot lie we are losing people on medication especially those we put on medication during the period of test-and-treat because they were not ready (KII 08 DHO HIV Program Manager 01).

The policy makers on the other hand seemed to have ignored the potential resistance to the test-and-treat-all policy from the public. They took it for granted that community members would appreciate this policy because of its benefits as one policy maker recounted.

It was aimed at being curative and preventive, that all people found positive should be given ARVs for free. Even testing is free and preventive because we know that those on HIV medications will stop spreading the infection if they comply with treatment. From the numbers the policy is doing wonders a lot of people are on drugs now (KII 15 Policy maker MoH 03).

##### Unintended consequences of the HIV/AIDS policy change to test-and-treat-all

According to the participants, there were unintended consequences on the health system resulting from the HIV policy change to test-and-treat-all. The emergent themes describe the unintended consequences on human resources for health and facility infrastructure.

**Human resources and infrastructure adjustment**

The frontline health providers and managers described how the test-and-treat-all policy change affected the operations of the health systems from the primary, secondary and tertiary levels of health care. Some of the changes brought about by the policy change included increased demand for human resources, infrastructure adjustment to accommodate the expanded services, changes in work schedules and increased working hours. A managerial health provider had this to say.

It changed the way we provide services because it meant that services were now tailored towards addressing that policy. But it was not easy on the ground because the staff and buildings remained the same. So, workload increased because as a clinician, as someone is attending to clients when you find a positive, you must shift certain things in the facility so that they receive the treatment there and then (KII 06 DHO manager).

## Discussion

The results revealed that to communicate the test-and-treat-all policy change, the government relied on formal and informal channels to relay information to the health providers. Whilst HIV/AIDS policy changes are reflected in the National AIDS Strategic Framework, there was inadequate awareness of this policy among the health providers. The use of informal communication channels such as verbal and text instructions affected health providers’ implementation of the test-and-treat-all policy change. Electronic and print media were ineffective in communicating the test-and-treat-all policy change to some sections of the public. The implementation of the test-and-treat-all policy change was affected by the top-down stakeholder engagement, limited health worker training, and poor financing. Acceptability of the test-and-treat-all policy change was shaped by positive health provider perceptions of its benefits, limited sense of policy ownership, and resistance by the non-treatment ready patients. Furthermore, they were unintended consequences of the test-and-treat-all policy change on human resources for health and facility infrastructure. 

### Communication of the HIV/AIDS policy change to test-and-treat-all

The use of print media like newspapers, posters and brochures to communicate the test-and-treat-all policy change to the public was found to be ineffective. For example, newspapers were unlikely to be read by the younger population, who have a significant burden of HIV/AIDS in Zambia. Though electronic media such as radio and television have a wider reach, they are still inaccessible in most remote parts of the country. On the contrary, other studies assessing public knowledge of HIV/AIDS in similar settings have found that television and radio remain the main source of information [25, 26]. Further, although IEC materials such as posters and brochures with HIV/AIDS policy change messages were widely disseminated with the support of international partners, poor literacy and inability to comprehend inhibited communication of the test-and-treat-all policy change with the public. Similar findings have been reported in other studies [27–29].

With regards to communicating the test-and-treat-all policy change to the health providers, the study revealed that there were no clear HIV policy communication guidelines in the health facilities, and this was left to the discretion of policy makers and senior management. The health providers did not refer to the National AIDS Strategic Framework, but rather indicated that the instructions regarding the HIV policy change to test-and-treat-all were given to them by their superiors. These instructions were sometimes communicated to the health providers through informal platforms such as WhatsApp. Such methods of communicating health policy change may explain why the test-and-treat-all was considered as 'mandatory testing' during the initial phases of implementation, as every health provider understood it differently [30]. Similarly, another study found that failure to properly communicate policy change with implementing agents may not only result in policy misinterpretation, but also, inertia to implement key policy change activities [16]. The unclear mechanisms of communicating health policy change and use of various channels may be responsible for distorting policy intentions and generating different sentiments towards the test-and-treat-all strategy among the health providers.

### Implementation of the HIV/AIDS policy change to test-and-treat-all

The introduction of the test-and-treat-all policy change in Zambia was said to be so sudden that most of the frontline health providers were caught unaware. Immediately, the policy change was announced by the president, the policy makers and international partners started to actively promote it through advertisements on various media platforms. However, the test-and-treat-all policy change was not in alignment with the existing HIV service capacity in the health facilities in terms of infrastructure, human and material resources [31]. For example, the frontline health providers were affected by the increased workload to accommodate the test-and-treat-all policy change. Our findings are similar to those of studies done in Uganda which found that policy change without corresponding investment in human and material resources may jeopardize service delivery [32, 33].

Another important factor that affected the implementation of the test-and-treat-all policy change was the limited financing allocated to HIV health services which has also been reported elsewhere [34]. Testing and treating all HIV patients without having to wait for the reduction in their CD4 count entails having adequate resources for basic HIV/AIDS testing and treatment supplies [31]. However, this was not the case as some of the health providers reported stock out of basic HIV testing and treatment supplies. A costing study found that close to 80% of HIV funding in Zambia is supported by the donor community [34], which can potentiallly undermine the long-term sustainability of the test- and-treat-all policy change.

There was a limited sense of ownership of the test-and-treat-all policy change among the health providers. This was due to the perception that the test-and-treat-all policy change was adopted to attract more donor funding to the HIV/AIDS programme in Zambia. Policy ownership among the health providers is important for them to not only appreciate but also sustain the intentions of the policy change [14]. Enhancing policy ownership can be done through empowering the health providers with knowledge and early involvement in the policy processes including formulation of appropriate communication strategies [14]. However, another study warns that over-consultations could derail policy change especially when dealing with a public health problem of such magnitude as HIV/AIDS that requires urgency [35]. This may explain the quick presidential announcement of the test-and-treat-all policy change as opposed to taking a more measured stages heuristic approach to policy change.

There was resistance to the test-and-treat-all policy change by the patients because they misinterpreted it as a mandatory HIV testing. Whilst some health providers provided counselling to clients, others seemed to make anti-retroviral treatment compulsory for newly HIV-diagnosed patients. However, not all clients accept mandatory testing and treatment when not ready to commence treatment. This approach by some health providers is contrary to the human rights approach to healthcare provision which underscores that consent to HIV testing and treatment is fundamental and should be treated with autonomy for every individual [36]. 

### Study strengths and limitations

This study had several strengths. Firstly, the interviewing of different categories of participants—the policy makers, managerial health providers and the frontline providers—did not only give a rich perspective but also enabled the triangulation of data. This also increased the trustworthiness of our findings [37]. Secondly, we believe the iterative reading, analysis and discussion of the emergent themes to develop the data code list within the research team enhanced the reliability of the findings [38]. However, we noted a few limitations. The gathering of information from only the health providers perspective does not give a complete picture of how the public perceived the communication of the test-and-treat-all policy change. Furthermore, the assumption that the study participants are knowledgeable about all aspects of the test-and-treat-all policy change may not completely be accurate. We are cognizant of the recall bias regarding some aspects of the policy communication process that the participants may not completely have understood, and the time that may have elapsed since the policy was implemented. Lastly, even with some of these limitations, we believe this study contributes valuable knowledge to an immensely under-researched area of health policy and systems research.

## Conclusion

The main objective of the test-and-treat-all policy is to eliminate HIV/AIDS in Zambia by the year 2030. However, communication of the test-and-treat-all policy change was ineffective leading to policy misinterpretation and resistance among the health provider and patients. Our findings highlight the need to address various factors including strengthening policy change dissemination and awareness among health providers and HIV patients, as well as, addressing context-relevant implementation factors that impact the policy’s success. There is a need for more collaboration among stakeholders to enhance communication and devise strategies that promote successful implementation and sustainability of the test-and-treat-all policy change.

## Data Availability

The field notes and interviews recorded verbatim and transcripts generated during data collection and analysis of this study are not publicly available to ensure the confidentiality and anonymity of the participating organizations and participants but can be accessed on request.

## Abbreviations

HIV: Human immunodeficiency virus
HCWs: Health care workers
T&T: Test and treat
NAC: National HIV/AIDS/STI/TB Council
MoH: Ministry of Health
